# Real-Time Fault Detection and Condition Monitoring for Industrial Autonomous Transfer Vehicles Utilizing Edge Artificial Intelligence

**DOI:** 10.3390/s22093208

**Published:** 2022-04-22

**Authors:** Özgür Gültekin, Eyup Cinar, Kemal Özkan, Ahmet Yazıcı

**Affiliations:** 1 Department of Informatics, Eskisehir Osmangazi University, Eskisehir 26040, Turkey; ogultekin@ogu.edu.tr; 2 Center of Intelligent Systems Applications and Research (CISAR), Eskisehir Osmangazi University, Eskisehir 26040, Turkey; kozkan@ogu.edu.tr (K.Ö.); ayazici@ogu.edu.tr (A.Y.); 3 Department of Computer Engineering, Eskisehir Osmangazi University, Eskisehir 26040, Turkey

**Keywords:** autonomous transfer vehicle, deep learning, edge artificial intelligence, FIWARE, real-time condition monitoring

## Abstract

Early fault detection and real-time condition monitoring systems have become quite significant for today’s modern industrial systems. In a high volume of manufacturing facilities, fleets of equipment are expected to operate uninterrupted for days or weeks. Any unplanned interruptions to equipment uptime could jeopardize manufacturers’ cycle time, capacity, and, most significantly, credibility for their customers. With the help of smart manufacturing technologies, companies have started to develop and integrate fault detection and classification systems where end-to-end constant monitoring of equipment is facilitated, and smart algorithms are adapted for the early generation of fault alarms and classification. This paper proposes a generic real-time fault diagnosis and condition monitoring system utilizing edge artificial intelligence (edge AI) and a data distributor open source middleware platform called FIWARE. The implemented system architecture is flexible and includes interfaces that can be easily expanded for various devices. This work demonstrates it for condition monitoring of autonomous transfer vehicle (ATV) equipment targeting a smart factory use case. The system is verified in a designated industrial model environment in a lab with a single ATV operation. The anomaly conditions of the ATV are diagnosed by a deep learning-based fault diagnosis method performed in the Edge AI unit, and the results are transferred to the data storage via a data pipeline setup. The proposed system’s Edge AI solution for the ATV use case provides significant real-time performance. The network bandwidth requirement and total elapsed data transfer time have been reduced by 43 and 37 times, respectively. The proposed system successfully enables real-time monitoring of ATV fault conditions and expands to a fleet of equipment in a real manufacturing facility.

## 1. Introduction

The emergence of the Industry 4.0 concept has changed the dynamics of many industrial fields, especially manufacturing. Old-fashioned manufacturing approaches and methods have become smarter with the advancement of industrialization and the information technologies such as big data, the Internet of Things (IoT), smart measurement techniques, and robotics. The integration and automation of the manufacturing processes via such technologies have contributed to developing more controllable and sustainable production environments [1]. Additionally, such advancements allowed production environments to become more flexible and efficiently designed, and operate with less human intervention. However, humans should not be simply substituted by artificial intelligence (AI) and automation on the shop floor; their man-hours should be utilized in more complicated tasks by employing smartly designed solutions [2]. Human operators in smart manufacturing environments are expected to focus on more creative and complex tasks, while repetitive tasks are generally assigned to intelligent systems like autonomous robots [3]. In today’s smart manufacturing environments, many autonomous devices accomplish routine tasks such as patrolling or transporting materials and parts precisely and safely without any human intervention. Additionally, they can also operate in places where human workers are restricted to work due to potential risks [4]. Therefore, they can operate repetitively even if the task is too dangerous or risky while keeping human workers secure [5]. Thus, this autonomous equipment has become an integral part of the equipment fleets in manufacturing facilities, and their uninterrupted operation and maximum uptime should be satisfied.

Intelligent devices like robots utilized in manufacturing environments are physical systems with varying degrees of autonomy that operate together in various and dynamic physical environments [6]. Since such robots are autonomous and expected to operate under different or unforeseen conditions, they are prone to different types of mechanical and operational faults. To continue operations and minimize unexpected downtime, the faults occurring while such devices are operating have to be detected, diagnosed and monitored on time. Thanks to the advancements in smart measurement technologies, computational capability of hardware systems, and the field of deep learning (DL), data-driven fault diagnosis methods in manufacturing environments promise great potential [7,8]. In data-driven fault diagnosis approaches, anomalies in equipment operations are diagnosed from the extracted features of measured sensor signals. A diagnostic decision is made regarding a healthy baseline state [9]. Sophisticated measurements via smart sensors can provide accurate data about the environment where the machines are operating in. Therefore, analyzing such data in an appropriate manner provides valuable information to help human operators to monitor components and trace the condition of the devices in real-time.

Effective diagnosis and monitoring can be made in environments where all equipment communicates with each other and other manufacturing operation systems within the network [10]. In other words, every component of the production environment should be evaluated as an IoT device and connected with the surrounding IoT devices. Thus, such devices are interconnected and constantly exchange data in real-time via virtual networks such as the cloud [11]. However, in many cases where real-time data transfer and analysis are essential, the bandwidth used by numerous devices and the computing workload of the centralized cloud can create bottlenecks due to the intensity of the data. Therefore, it is impractical to move all of the raw data to the centralized cloud server to process. One of the potential solutions for the bottlenecks mentioned above is edge artificial intelligence (edge AI), which combines edge computing and AI. Edge AI offers preprocessing or inference from the data acquired via smart sensors attached to IoT devices at the edge nodes, close to the source, before sending it to the centralized cloud. This approach decreases the volume of the data required to transfer and provides low latency, faster responses, data security, and scalability [12]. Consequently, edge AI provides practical solutions for industrial IoT devices like autonomous transfer vehicles (ATVs), which are susceptible to faults, rely on high-speed data rates and low latency transmission, and require real-time condition monitoring.

Robots are a vital part of manufacturing, and they need to be monitored with software systems to alleviate sustained manufacturing operations similar to other process equipment. In this work, we present a generic real-time condition monitoring architecture and a system implementation that can be adapted for a fleet of industrial ATVs to overcome the problem mentioned earlier. Uniquely designed edge processing algorithms utilizing sensor fusion and DL offer significant real-time advantages for sensor preprocessing and edge AI approaches. A new testbed, which consists of an industrial ATV, an edge AI accelerator, a customized open-source data distributor middleware platform utilizing FIWARE, a data storage unit, and a visualization tool, has been designed. The proposed system is generic and can be adapted to any manufacturing tools equipped with sensors and IoT communication protocols. The platform solution is specifically designed to handle condition monitoring of the robotics fleet in our ATV use case. The main contributions of this paper are:

- A generic real-time fault diagnosis and condition monitoring architecture utilizing Edge AI has been presented;
- The architecture is tested on industrial ATV use cases to diagnose operational faults;
- The data-driven DL algorithm utilizing sensor fusion is used on an edge AI accelerator hardware for inference;
- Real-time inference results are transferred from edge AI devices to the data storage utilizing a data pipeline enabled by a customized open source middleware platform called FIWARE.

The rest of the paper is organized as follows: Section 2 introduces a background and a literature review on related work for real-time diagnosis and monitoring, Section 3 describes the methodology, components, and details of the proposed architecture, and Section 4 presents the experimental setting and the results obtained from our approach. Lastly, the main conclusions and the future work are mentioned in Section 5.

## 2. Background and Literature Review

The Internet, IoT, and smart measurement technologies enable the transfer of physical devices or environments into the digital world. However, it is not easy for these devices and environments, composed of various hardware and other components, to communicate and interact efficiently in most cases. To provide such adaptation, some standards and applications have been developed. The FIWARE platform, which is one of them, can provide effective communication between IoT devices via its components. Many papers in the literature utilize the FIWARE platform to design smart environments effectively. For example, Celesti et al. [13] utilized the FIWARE platform to evaluate the design of the cloud and IoT-based health system services and applications composed of various remote medical sensors communicating with FIWARE platform components. López-Riquelme et al. [14] developed a software architecture using the FIWARE platform for precision agriculture and validated the architecture in a real agricultural environment for irrigation tasks. Fernández et al. [15] proposed a platform that offers an architecture utilizing the data collected from port measurement systems, IoT technologies, and the FIWARE platform to monitor and make decisions for seaport environments. Araujo et al. [16] evaluated the efficiency of the FIWARE platform on smart city solutions by developing a new testbed and emulating large-scale IoT deployments. The research performed in different areas show that the FIWARE platform and its components provide an effective and secure communication environment for various IoT devices. Thus, the FIWARE platform is chosen to develop a flexible and generic framework in this work.

Most data-oriented smart solutions offer an environment where IoT devices actuate and collect data from the environment while a centralized server processes the acquired data. However, this approach can have some drawbacks related to data security, excessive bandwidth usage for the network, and latency. Several studies utilizing edge AI technologies are performed in the literature to overcome such drawbacks. For example, Li et al. [17] provided a low-latency edge AI framework named Edgent using deep neural networks for inferencing at the edge. Key et al. [18] evaluated the feasibility of edge processing techniques based on single-shot multibox detectors on smart parking surveillance tasks via real-time video regarding flexibility, detection accuracy, and system reliability. Huang et al. [19] proposed an edge AI framework based on a digital twin for anomaly detection and real-time condition monitoring of industrial systems. The framework is tested for the LiBr absorption chiller, commonly used equipment for air conditioners, for fault diagnosis. Shah et al. [20] proposed an edge AI framework for real-time event detection utilizing lightweight convolutional neural networks (CNNs) and multi-sensor data fusion techniques. The proposed framework tested with a benchmark air quality dataset with two different DL models. Even if there are several studies about edge AI techniques and frameworks, most of them are not verified in a real testing environment, or they are domain-specific. Therefore, there is still a requirement for a generic framework that can be easily adaptable to many use case scenarios in the field.

With the advancements in sensor and measurement technologies, industrial equipment utilization capacity and lifetime can significantly increase. The data acquired from various sensors are processed further via signal processing techniques to enable further applications such as condition monitoring and fault detection of equipment while operating [21]. With the widespread use of machine learning in many fields, industrial applications also employ data-driven modeling techniques for fault detection and classification. These applications mainly benefit from DL techniques that generate high-capacity models by simply learning directly from raw data. There are many papers related to data-driven anomaly detection, fault diagnosis, and condition monitoring of IoT devices utilizing various sensors and DL techniques [22]. For example, Chen et al. [23] employed wavelet transform of vibration signals acquired via acceleration sensors as an input of their proposed DL model to identify the fault conditions of planetary gearboxes. Yao et al. [24] proposed a gear fault diagnosis approach based on time and frequency domain sound signals and DL in another work. Additionally, since different sensors can perceive the environment with different aspects, a fusion of multiple signals can help diagnose and monitor industrial machines’ conditions more accurately and robustly. For example, Wang et al. [25] proposed a DL method that utilizes the fusion of vibration and sound signals to diagnose faults of the bearings. Our recently published study [26] utilized time-frequency domain representations of vibration and current signals as the input of our proposed novel DL method to diagnose rotating machinery faults. Both studies justify that the fuse of multisensory signals information in an appropriate manner outperforms the results acquired by single sensor experiments in terms of accuracy, reliability and robustness. As a result, the generic framework proposed in this paper benefits from data-driven fault diagnosis approaches and is tested on a use case targeting a real industrial environment by employing DL algorithms with multisensory fusion approaches.

## 3. Proposed Method

This section identifies the elements of our proposed generic architecture. Firstly, the ROS platform, which is frequently utilized on robot hardware and other IoT devices, is briefly explained. Then, the concept of edge AI and its benefits are introduced. After this, the FIWARE platform, its main components, and how it works are briefly mentioned. Finally, the proposed approach for real-time fault diagnosis and condition monitoring is presented.

### 3.1. Robot Operating System

Mobile robots such as ATVs and other IoT devices generally consist of various hardware components such as engines, wheels, cameras, and other sensors. The heterogeneity of such components hinders the development of efficient and flexible software interfaces to control and employ robots. In addition to this, intercommunication for IoT entities can also suffer from such limitations. However, ROS middleware is designed to eradicate these barriers and provides a standardized development platform that includes common sensor driver and interface solutions to manage robot hardware with many sensors and actuator parts. The ROS provides an environment where distinct robots with similar components can benefit from the same code blocks with only minor modifications [27]. ROS is a collection of program packages that facilitates robots’ control and operations. ROS offers four fundamental concepts: nodes, messages, topics, and services [28]. The nodes are software modules that perform computations and communicate with each other via messages. A node can publish a message over a topic, and any node can subscribe to that topic to receive published messages. The service model is analogous to classical web services as client node requests and server node responses. Additionally, in a ROS platform, every node is required to connect to the master node in which all communication is planned and managed.

### 3.2. Edge Artificial Intelligence

The term “edge” is defined as any computation or network resources between the data sources and cloud servers in a system [29]. Generally, edge devices are small, decentralized, require low power to work, and are located close to data sources. Edge AI (or edge intelligence) is considered the combination of edge computing and AI. This combination can be explained as the use of computing resources of the edge nodes to process data acquired from hardware devices with machine learning or DL algorithms locally. Therefore, edge AI brings artificial intelligence and computational capacity near data sources. Instead of sending whole raw data collected from the hardware of IoT devices to the centralized cloud servers, only the results of inference models performed on the edge nodes are sent. Since acquired data is processed in decentralized edge nodes, network bandwidth usage and computational resources belonging to centralized servers will decrease. Thus, utilizing edge AI techniques decreases communication costs and latency and increases reliability, scalability, data security, and privacy [30].

### 3.3. The FIWARE Platform

The FIWARE platform, a middleware, facilitates the development of smart manufacturing solutions by its components called generic enablers (GEs). GEs are software components developed for various applications and/or architectures. The idea behind the platform is to combine proper GEs based on the requirements of a specific system. A context broker (CB) is the core component of any FIWARE solution [31]. Other GEs are application-specific and deal with tasks like interfacing with IoT and robots, managing context data/API, and processing, analyzing, and visualizing context information. The simplest FIWARE platform consists of a CB and a database to store context data. In IoT scenarios, CB handles the context entities that include the information generated from IoT devices. Additionally, FIROS, another GE, establishes communication between the robotic domain and cloud to transform ROS messages into NGSI v2 entities that can be utilized and published by FIWARE CBs.

### 3.4. The Proposed System Architecture

**Figure 1:**
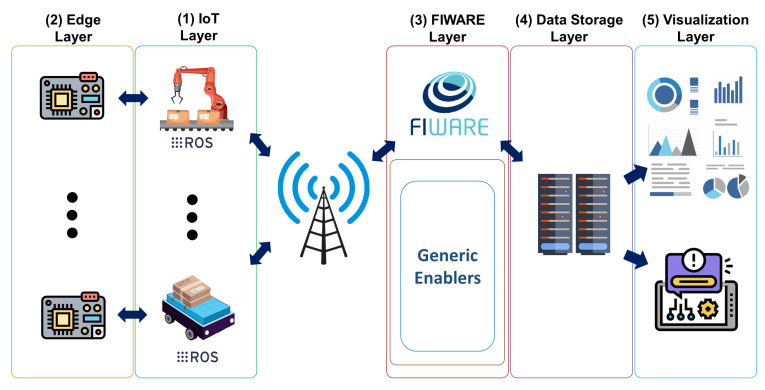
The proposed system architecture.

This paper proposes a generic real-time fault diagnosis and condition monitoring architecture utilizing edge AI. The architecture is composed of five main layers: (1) the IoT layer, (2) the edge layer, (3) the FIWARE layer, (4) the data storage layer, and (5) the visualization layer. (1) The IoT layer consists of several devices with ROS running inside. These devices collect data via the sensors mounted. The ROS topic publishing mechanism acquires data over pre-determined topics with proper messages through the master node. (2) The edge layer includes edge AI devices that can perform DL algorithms for inference. Since they are small devices and decentralized, each is located close to one of the IoT devices in the IoT layer. In addition, each edge device is connected to the IoT device with an Ethernet cable and subscribes to the topics publishing from the assigned IoT device via ROS. The real-time sensor data acquired from topics are used in the DL-based fault diagnosis algorithm for inference, and the results are published over another topic on the master node. (3) The FIWARE layer consists of GEs as a CB, a database, and FIROS. FIROS transforms the message inside the topics published from the master node to NGSI v2 entities and sends them from IoT devices to the CB. Then, such entities and attributes are published from the CB and stored in the database. (4) The data storage layer is mainly composed of data persistence components such as a database server and a processing engine that can convert the data published from CB to the appropriate data format to store in the database. This layer gets the data from the FIWARE platform and stores it. (5) The visualization layer consists of tools that can create dashboards from the data stored in the database server to monitor the condition of IoT devices in real-time. This layer provides interaction between end-users and computers and helps to make decisions about the monitored entities and the overall system. The proposed architecture is illustrated in Figure 1.

### 3.5. Theoretical Background

The most significant contribution of the edge AI technology in the proposed approach is enabling AI models to be performed in an edge unit that is closer to the data source. In this paper, the short-time Fourier transform (STFT) and the LeNet-5 CNN model have been implemented on the Edge AI unit for the ATV use case. STFT transforms any measured non-stationary signal into the time-frequency domain, and the transformed representation of the signal consists of both time and frequency information. STFT slices the waveform to short time segments by a windowing function and then applies a standard Fourier transform (FT) on each segment to transform them into the time-frequency representations called spectrograms. The basic operation formula is defined as

(1) $$STFT_{x}(\tau ,f)=\int_{-\infty }^{+\infty }x(t)h(t-\tau )\exp^{-j2\pi ft}dt$$

where $x\left(t\right)$ is a segment of the measured signal, and $h\left(t-\tau \right)exp^{-j2\pi ft}$ is the basis of the STFT function. On the other hand, the LeNet-5 model is preferred for the use case due to its simplicity, efficiency in computational cost, and inference performance for real-time fault diagnosis. The LeNet-5 model consists of seven layers: convolution, pooling, convolution, pooling, convolution, fully connected, and classification layers, in order [32]. The convolution operation formula is defined as

(2) $$y_{j}=f\left(\sum_{i}X_{i}*W_{ij}+b_{j}\right)$$

where $W_{ij}$ maps the convolution kernel corresponding to the ith input feature map ($X_{i}$) to the jth output feature map ($y_{j}$) with the convolution operator $\left(*\right)$ before adding the bias parameter ($b_{j}$) and applying the nonlinear activation function (f). The ReLU function described in Equation (3) is used as a nonlinear activation function in each convolution operation.

(3) $$f(x)=\left\{\begin{array}{cc} x & x>0\\ 0 & x\leq 0 \end{array}\right.$$

Pooling layers are used to extract quadratic features and reduce the dimensionality of feature maps. Maximum pooling operations with a 2 × 2 kernel, which can half-size each feature map, are used for the LeNet-5 model utilized in this ATV use case. Each neuron in the output of the last convolution layer is connected to the fully connected layer with the dense operation. In the output layer, dense neurons at the fully connected layer are classified with the Softmax classifier to make the final decision. Softmax is a widely used and effective classification function, especially for multi-classification tasks.

## 4. Experiments

This section details the industrial ATV use case scenario utilizing the implemented real-time fault diagnosis and condition monitoring architecture with the edge AI techniques. Firstly, the system overview and device components are explained. Then, the experimental setting used in test scenarios is presented in detail.

### 4.1. System Overview and Device Components

The efficiency of the proposed architecture is tested in an environment in which a real industrial workspace is mimicked as much as possible. However, the proposed system is not yet deployed in real-world manufacturing. The current architecture assumes that it will be integrated without major modifications to the system entities. Additionally, even if the experiments are performed utilizing a single ATV, the system is expected to scale for a fleet of ATV equipment and various other IoT devices for condition monitoring. Any equipment on which ROS is running is assumed to be integrated into the system smoothly. Moreover, the DL model used at the edge for inference is assumed to be a static one, and there is no auto-model update mechanism in the system implemented yet. Therefore, the long-term robustness of the edge model for equipment state drifts is not tested and assumes that the edge model can be updated manually by a human operator when needed.

The ATV with ROS middleware is used as an IoT device in the experiments. The internal hardware and software prevent ATVs from unexpected collisions that are possible while performing given tasks. The utilized ATV is 1026 mm in length, 728 mm in width, and 325 mm in height and has two main wheels of 200 mm and four casters with 80 mm diameters. There are two identical DC motors for each main wheel, and there are also two groups of sensors attached to those motors to measure temperature, humidity, sound, vibration, and velocity. These smart sensors perceive the environment and acquire the data according to the specified data rate. Additionally, the acquired data are published over the ROS middleware and FIROS. Therefore, an edge device connected to the ATV can subscribe to the measured data. Moreover, the ATV has an onboard Ethernet card, WLAN, and Bluetooth for communication.

The NVIDIA Jetson TX2 GPU module is used as the edge AI device for this work. Since the module has an onboard Ethernet card, has a size of 170 mm length × 170 mm width, and requires low energy, it is placed inside the ATV’s bodywork. The board has 256 NVIDIA CUDA Cores and 8 GB 128-Bit LPDDR4 Memory, enabling it to perform DL algorithms fast and effectively while only requiring 19 V to work. Therefore, it is possible to make real-time inferences from the sensor data published by the ATV. There are also server computers virtualized to run Orion CB, MongoDB, data storage, and visualization tools containers. The server is connected to the same network as the ATVs and can communicate with them via an open-source microservice called FIROS GE installed on ATV onboard computers. The main workstation has an Ubuntu 20.04 operating system, two Intel Xeon Gold 5218R CPU with 40 Cores, and 64 GB DDR4 RAM.

### 4.2. Experimental Setting

**Figure 2:**
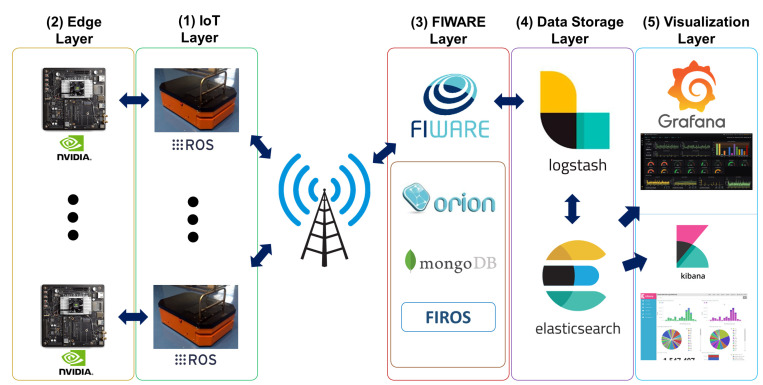
The ATV use case for proposed generic architecture.

Since the proposed architecture is a generic one, it is possible to scale to a multi-robot ATV fleet use case and expand into other types of hardware entities with custom data adapters. The experimental setting is designed as a real industrial environment for the ATVs and is demonstrated in Figure 2. In the IoT layer, there is an ATV with Ubuntu 16.04 operating system and the ROS middleware. The ATV is connected to the Internet with a Wi-Fi connection and can operate autonomously for tasks such as wandering or load carrying. While the ATV performs intended tasks, sensors attached to the ATV body provide real-time data about the perceived workspace. Via ROS middleware, the data collected from ATV sensors is published over separate topics as messages at a specified data rate. Therefore, any device communicating with the ATV can subscribe to that topic and consume them.

**Figure 3:**
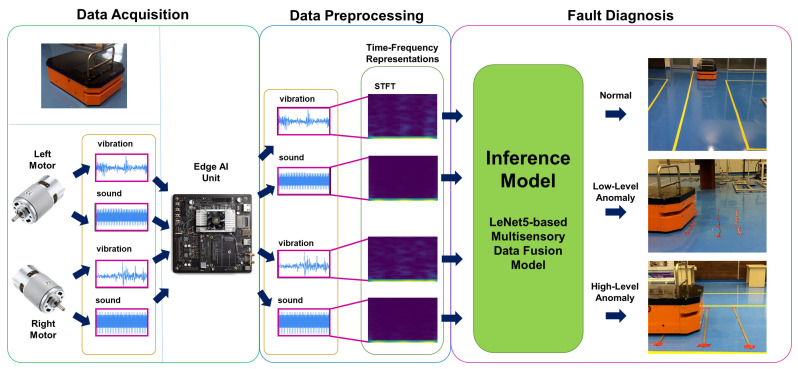
The real-time multisensory fault diagnosis inference model was performed on the NVIDIA Jetson TX2 GPU module.

An NVIDIA Jetson TX2 board is utilized as an Edge AI device in the edge layer. The board has an Ubuntu 18.05 operating system and the ROS middleware as well. Since the board is located inside the bodywork of the ATV, the required energy to work is ensured by the battery of the ATV, and a cat6 Ethernet cable provides the connection between both devices. The board has a strong GPU unit and can handle DL models and real-time inference. A DL-based real-time operational fault diagnosis method utilizing sensor fusion is used for the inference in the experiment [33]. This method is tested on the offline data collected from the ATV and provides significantly high accuracy of over 98.61% at each of 25 trials. The method obtains the sound and vibration data of two DC motors as input, then preprocesses them with the STFT, transforming non-stationary signals to the time-frequency domain to obtain spectrograms. After preprocessing with specific parameters, spectrograms with the size of 64 × 64 were acquired for each signal of 512 data points. Then, the LeNet-5-based sensor fusion model is fed by four spectrograms obtained synchronously and diagnosed for three different operational fault conditions. These operational conditions are normal conditions (there is no obstacle or intervention), low-level anomaly conditions (4 mm copper cables used as obstacles), and high-level anomaly conditions (identical metal bumpers fixed to the ground as obstacles). For each of the three operational conditions, sensory data is collected at 100 Hz, while the ATV is repeatedly operating under the task of moving straight for 3.5 m in the same testing environment. After this model is trained and learned with the 80 samples for each condition, the model is moved to the Edge AI unit to make inferences in real-time. Figure 3 represents the real-time inference model performed on the NVIDIA Jetson TX2 board.

As the figure depicts, data acquired from microphones and IMU sensors attached to both motors are published in separate ROS topics. Then, the edge AI board subscribed to that topic and preprocessed the data via STFT with appropriate parameters to obtain spectrograms. After this, each synchronous spectrogram is fed into the model trained to make an inference. The inference and utilized data results are published in other separate topics over the master. Therefore, these are available for other IoT devices and FIROS GE. In addition to this, even if subscribed data are preprocessed as batches of 512 data points, a sliding window shifting 128 data points is used to diagnose operational conditions in real time more accurately. Meanly, each successive input has 384 similar values. Since the data collection frequency is 100 Hz, each successive real-time inference can be performed in 1.28 s.

In the FIWARE layer, three GEs are used for the ATV use case: Orion CB, MongoDB, and FIROS. Orion CB is the broker that provides the FIWARE NGSI v2 API to perform operations like a subscription on context information. MongoDB is a database to store NGSI entities and their attributes provided by Orion CB. FIROS establishes communication between the IoT and FIWARE layers by transforming ROS messages into NGSI entities and sending them to the Orion CB. FIROS is placed in the ATV (master node) to transform published topics into NGSI entities for such scenarios. Therefore, the topics, including real-time analysis results and data utilized for inference, are transferred to the Orion CB via FIROS over the Wi-Fi connection established. Then, Orion CB performs operations like publishing NGSI entities and their attributes while MongoDB stores them.

Logstash is used as the processing engine in the data storage layer, and Elasticsearch is used as the data storage. Logstash is a data processing pipeline that consumes the data, transforms it to the intended format, and sends the outputs to Elasticsearch with real-time pipelining capabilities. On the other hand, Elasticsearch is a kind of data storage that stores the data to search and analyze rapidly. For the ATV use case, Logstash gets the data from Orion CB, converts it to JSON format, and sends it to Elasticsearch to store.

The visualization layer consists of Kibana and Grafana visualization tools for the use case. Kibana is a powerful visualization tool and naturally integrated with Elasticsearch. Grafana is another option that can be integrated into several data sources to analyze the data, create real-time dashboards, and alert depending on thresholds. Even if both tools have similar aspects, there are some significant differences. While Kibana is much more powerful for querying and searching the data, Grafana has a talented built-in alerting engine. The engine lets users specify conditional rules for dashboard panels to trigger alerts and notify users with various methods. For the use case, Kibana is used for querying and searching. At the same time, Grafana is used to create dashboards to monitor the data utilized and ATV conditions in real-time and alert decision makers if required.

## 5. Results and Discussions

This section describes the experimental results obtained while the ATV operates in an environment where various obstacles are fixed to the ground that can cause low or high-level anomalies. Firstly, the testing environment and real-time inference results are detailed. Then, the interface of the data storage is given. After that, the monitoring system and dashboards created are demonstrated. Finally, the obtained results are discussed in detail.

### 5.1. Testing Environment and Real-Time Inference

**Figure 4:**
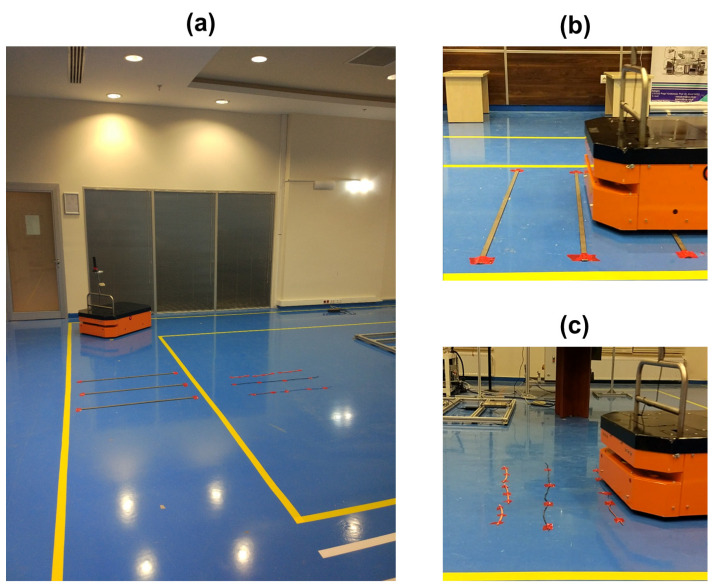
Testing environment and real-time inference: (**a**) testing environment; (**b**) obstacles for high-level anomaly, and (**c**) obstacles for low-level anomaly.

While the ATV is executing a predefined load-carrying task over a route, the edge AI unit makes inferences about the condition of the ATV utilizing the pre-trained DL model described in Figure 3. The client code, developed in Python and running on the ATV edge AI unit, is used to subscribe to the ROS topics, including the sound and vibration data of both motors published over the ROS master node, and run the DL model to make inferences. Figure 4a shows the simulated testing environment. In this environment, the ATV first passes over three metal pieces that are defined to cause high-level anomalies during the offline model training phase labeled from the starting point, as shown in Figure 4b. Then, the ATV path planning task directs the vehicle to continue to the path by following a rectangular shape route on the ground until it reaches the starting point coordinates. On its way back to the endpoint, the ATV also passes over three copper cables that are defined to cause low-level anomalies for the ATV operational conditions (Figure 4c). Note that the end-users can customize high and low-level anomalies in this study to any behavior during the specific factory environment conditions. While the ATV is operating the preprogrammed task, the data from sound and vibration sensors attached close to the DC motors are acquired. Then, these data are filtered and processed in the Edge AI unit to make real-time inferences about the condition of the ATV.

### 5.2. Interface of the Data Storage

**Figure 5:**
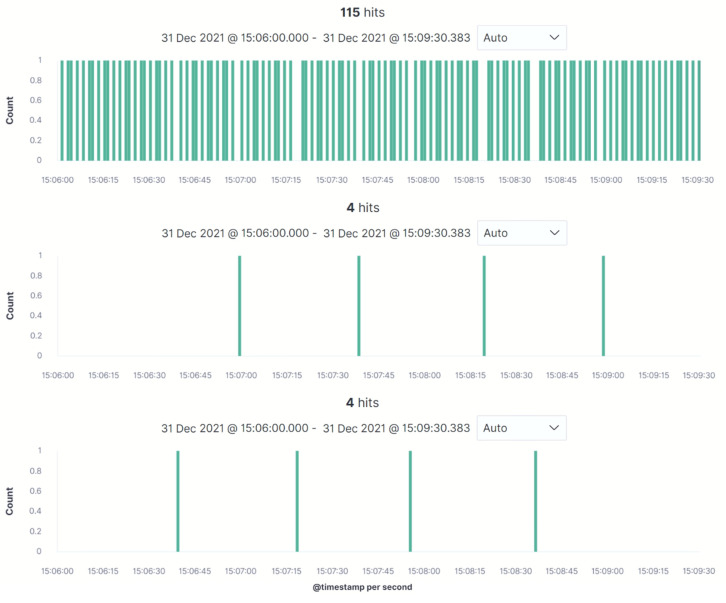
Visualization of the ATV’s condition data in Elasticsearch with Kibana.

The middleware platform transfers the data, implemented utilizing FIWARE and stored and managed to utilize the Elasticsearch data persistence stack. Each topic published by the ATV and subscribed by the FIWARE platform is listed in a separate index pattern at the data storage. The Kibana tool is used to discover and filter the data stored in Elasticsearch. Figure 5 provides an example of the results obtained while the ATV repeats a predefined load-carrying task four times in our testing environment. The chart in the first row represents the number of hits on the y-axis and its corresponding timestamps on the x-axis when the ATV is detected to be in normal operational condition. The middle graph represents the cases where the ATV is detected to be facing low-level anomalies. The bottom graph represents the cases where the ATV has faced high-level anomalies with corresponding timestamps on the x-axis. The ATV mostly operates in the field where there are no obstacles in the experiments. Therefore, the fused data obtained via sensors reflect the condition of the field successfully, as the majority of the data entry results in the dashboard show healthy hits. When the main wheels of the ATV interact with the metal bumpers attached to the ground, the obtained data are analyzed and classified as a high-level anomaly condition. Additionally, whenever the ATV passes over copper cables, this action seems to increase low-level anomaly hits since this physical condition was introduced to the model as a low-level anomaly. The time axis also points out the occurrences of anomaly instances and the high model capacity of the DL model. The timestamp information given in the dashboard figure is verified experimentally to match actual real physical occurrences of the anomaly events with minor delays due to network overhead. The time interval between low and high anomaly hits depends on the physical distance between the cables, the turning speed, and the pace of the ATV.

### 5.3. The Data Visualization and Alert Tool for Condition Monitoring

**Figure 6:**
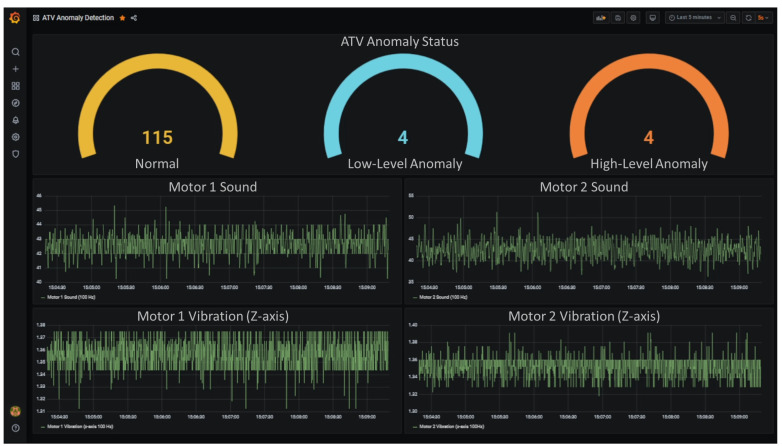
Grafana dashboards for real-time condition monitoring.

The results and the data used for the inference are consumed to create dashboards for real-time condition monitoring and decision making. Grafana is used to visualize data stored in Elasticsearch and alert for unintended data changes in real-time via sending an email to end-user stakeholders. Grafana is a flexible tool, and it can be customized easily for different data formats as well. Figure 6 shows the dashboard created for end-user operators to visualize the monitored environment data and the Edge AI inference results. The data presented in Figure 6 represents the last 5 min of an ATV operation and is refreshed every 5 s. The real-time condition of the ATV is represented with the gauge style chart, and the raw sound and vibration data obtained from both motors are presented in two-dimensional graphs. Since the data collection frequency is 100 Hz and each graph is updated every 5 min, 30,000 data points appear in each graph. Whenever the dashboard is refreshed, the lastly obtained 500 data points are appended to the right-hand side of each graph, and the older 500 data points are removed from the left-hand side. Since the inferences made by the DL model reflect the 512 data points at a time, the changes in anomaly hits are directly matched with the appended 512 data points. Therefore, the graphs below represent the sound and vibration data obtained from each motor and help analyze the difference between the data obtained from healthy and anomaly cases. In addition, such graphs are also helpful indicators to monitor the health status of hardware subscribed to the system.

### 5.4. Discussion

This study develops and tests a real-time fault diagnosis and condition monitoring system incorporating edge AI and a custom-developed open source middleware platform utilizing FIWARE. The proposed system architecture is generic, and it can be easily adapted and scaled for different use cases allowing the monitoring of the condition of many different IoT devices simultaneously. In addition, employing edge AI technologies has shown us experimentally that data processing utilizing edge resources can have power advantages. The edge processing approach offers significant performance contributions in latency, privacy, communication costs, and storage costs. To quantify the improvements provided by edge AI, various metric values are calculated. Table 1 lists the calculated metrics for two different scenarios such as (1) sending all of the raw sound and vibration data obtained from the sensors to the centralized server and processing them in it, and (2) filtering and processing the data at the edge AI unit and only transferring the DL model inference results through the centralized server for storing and visualizing. Three metrics are calculated and given in the table: average bandwidth usage, data transfer delay, and average duration for inference in both scenarios. The raw sensor data column represents the load caused by transferring all sound and vibration data acquired from sensors to the centralized server and processing it. On the other hand, processed data at the edge AI unit column shows the results of transferring processed data at the edge AI unit to the centralized server to store and visualize. Calculation of such performance metrics is crucial for real-time and safety-critical systems where various autonomous equipment and human operators have to work together in the same environment.

Since the raw data are preprocessed locally at the edge AI unit, it is not completely transferred to the centralized server. Only the inference results are transferred to the central server. This approach has dramatically reduced the use of network resources in terms of latency and bandwidth usage. In Table 1, the comparison of the required network bandwidth when all the sound and vibration data of two identical motors used for the DL-based fault diagnosis algorithm are transferred to the server, and the network bandwidth usage of the data obtained as a result of the analysis and filtering of the edge AI unit is given. The values in the table are the average values of 60 s, and as a result of the analyses performed with edge AI, it is observed that the need for network bandwidth has been reduced by approximately 43 times. In another calculation, the delays in the data transfer are compared. The delay here is due to data density, network infrastructure, and resources of the centralized server used for reading/writing the data. The total elapsed time for data transfer has been reduced by 37 times with the proposed approach. Considering the amounts calculated, it is seen that there are significant delays in the process of moving the data to the central server without processing on any edge AI unit. This would hinder the performance of real-time analysis if it were performed on a central server, especially for time-critical applications such as fault diagnosis. However, as can also be seen from the table, in the case of making inferences on the centralized server, although there is a speed advantage of 68 times, the delay experienced in data transfer overshadows this advantage. Furthermore, in the case of scaling the designed system to an ATV fleet operation, since an edge AI unit can be placed on each ATV in the designed architecture, in case the number of ATVs is increased 100 times, the resources in the central server would be required to increase linearly. However, the resource usage of the edge AI device will not change due to the distributed structure. As a result, edge AI technology in such scenarios appears to be much more practical than the traditional central server approach. Even if the advantages of the proposed approach outperform traditional ones, there are some drawbacks, such as hardware expenditures and expertise requirements, especially when the system is scaled for various and numerous IoT devices are not taken into account. Since the edge layer consists of stand-alone edge AI devices for each piece of equipment, the financial cost can be concerning for a large-scale system end-user. Therefore a current edge unit can be replaced with a more cost-friendly device. Therefore, the prototype system hardware expenditure analysis should be performed. Since ML automation tools are not yet integrated into the system, the proposed prototype system might still require some human-level expertise to maintain low-level technical operations. The end-users can work on this to bypass such challenges in the field.

## 6. Conclusions and Future Work

This study presented a generic real-time fault diagnosis and condition monitoring architecture utilizing edge AI and an in-house custom-developed middleware solution utilizing the FIWARE platform. Although we demonstrated the system in a use case for a smart factory solution with an ATV condition monitoring application, the architecture is a generic one and can be adapted to various use cases from logistics to agriculture, health applications, and security systems. Even if the presented architecture features ROS, it can also be adapted to various IoT devices supported by generic enablers and custom data adapters. The edge AI device uses the collected data via multiple sensors attached to ATV for inference. The utilized DL method is tested on the offline data collected from the ATV and provides significantly high testing accuracy with an average of 99.94% for 25 trials. Then, the results obtained and the data used are transferred to the data storage. It is easily possible to terminate raw sensor data transfer over the network by customizing sensor subscriptions through the middleware system. Finally, the visualization tool consumed the data to create dashboards and alert admins if required. To demonstrate the effectiveness of the implemented system, experiments were performed by making an ATV execute a predefined load-carrying task in a model factory environment where multiple operational anomalies are introduced. The results showed that the implemented system could diagnose and monitor conditions of ATVs in real-time. Additionally, the proposed edge AI solution for the ATV use case reduces the required network bandwidth 43 times. Total elapsed time for data transfer also decreases 37 times compared to traditional central server approaches. Although the experiments and the system’s viability are demonstrated utilizing a single ATV, it is also possible to expand the middleware layer with multiple IoT devices easily as multiple instances of the FIROS platform can be deployed to run across several ATV units. However, this also brings tradeoffs between the hardware expenditures and the number of available ATV units. Thus, the system can be integrated into real-world problems to contribute to increased operational efficiency in a smart manufacturing environment.

In future work, the scope of this paper will be extended in different aspects. Dynamic edge AI model training and update mechanism will be introduced in case of DL model performance drifts. Therefore, we will expand our architecture with a dynamic model approach and AutoML integration. By integrating AutoML techniques into the proposed architecture, model auto-update scenarios can be implemented smoothly. Moreover, our future work will also focus on testing the proposed architecture at a scale by integrating multiple and different kinds of equipment monitoring interfaces into the system.

## Figures and Tables

**Table 1:** Comparison of the metrics calculated from the raw sensor data and the processed data at the edge AI unit.

	Raw Sensor Data				Processed Data at the Edge AI Unit	
Metric	Motor 1 Sound	Motor 2 Sound	Motor 1 Vibration	Motor 2 Vibration	Inference Results	Filtered Sound & Vibration Data
Average Bandwidth Usage	61.62 KB/s	64.59 KB/s	14.78 KB/s	14.62 KB/s	17.55 B/s	3.63 KB/s
Data Transfer Delay	1238.54 s	1301.39 s	342.11 s	329.92 s	0.07 s	87.22 s
Average Duration for an Inference	0.00669 s *				0.4537 s **	

* Using the resources of a centralized server for a single ATV; ** Using the resources of an edge AI device.

## Data Availability

Not applicable.

## Abbreviations

The following abbreviations are used in this manuscript:

AI	Artificial intelligence
API	Application Programming Interface
ATV	Autonomous transfer vehicle
CB	Context broker
DL	Deep learning
Edge AI	Edge artificial intelligence
GE	Generic enabler
IoT	Internet of Things
JSON	JavaScript Object Notation
NGSI	Next-Generation Service Interfaces
ROS	Robot Operating System
STFT	Short-time Fourier transform
